# Aging-Induced Brain-Derived Neurotrophic Factor in Adipocyte Progenitors Contributes to Adipose Tissue Dysfunction

**DOI:** 10.14336/AD.2019.0810

**Published:** 2019-08-10

**Authors:** Hyun-Doo Song, Sang Nam Kim, Abhirup Saha, Sang-Yeop Ahn, Seun Akindehin, Yeonho Son, Yoon Keun Cho, MinSu Kim, Ji-Hyun Park, Young-Suk Jung, Yun-Hee Lee

**Affiliations:** ^1^College of Pharmacy, Yonsei University, Incheon, Republic of Korea.; ^2^College of Pharmacy and Research Institute of Pharmaceutical Sciences, Seoul National University, Seoul, Republic of Korea.; ^3^College of Pharmacy, Pusan National University, Busan, Republic of Korea.

**Keywords:** BDNF, adipose tissue, sympathetic innervation, adipocyte progenitors, aging

## Abstract

Aging-related adipose tissue dysfunction contributes to the progression of chronic metabolic diseases. We investigated the role of age-dependent expression of a neurotrophin, brain-derived neurotrophic factor (BDNF) in adipose tissue. Pro-BDNF expression was elevated in epididymal white adipose tissue (eWAT) with advanced age, which was associated with the reduction in sympathetic innervation. Interestingly, BDNF expression was enriched in PDGFRα^+^ adipocyte progenitors isolated from eWAT, with age-dependent increase in expression. In vitro pro-BDNF treatment caused apoptosis in adipocytes differentiated from C3H10T1/2 cells, and siRNA knockdown of sortilin mitigated these effects. Tamoxifen-inducible PDGFRα^+^ cell-specific deletion of BDNF (BDNF^Pdgfra ^KO) reduced pro-BDNF expression in eWAT, prevented age-associated declines in sympathetic innervation and mitochondrial content in eWAT, and improved insulin sensitivity. Moreover, BDNF^Pdgfra ^KO mice showed reduced expression of aging-induced inflammation and senescence markers in eWAT. Collectively, these results identified the upregulation of pro-BDNF expression in adipocyte progenitors as a feature of visceral white adipose tissue aging and suggested that inhibition of BDNF expression in adipocyte progenitors is potentially beneficial to prevent aging-related adipose tissue dysfunction.

Aging-related adipose tissue dysfunction contributes to the pathogenesis of chronic metabolic disease [1]. Adipose tissue aging can be characterized by several features, such as insulin resistance, alteration in mitochondrial metabolism, and inflammatory secretome profiles of adipocytes [2]. In addition to adipocyte intrinsic mechanisms, adipose tissue aging involves multiple micro-environmental components, such as adipocyte progenitors [3], vasculature [4], nervous input [5] and immune/stromal cells [6]. Several studies using rodent aging models demonstrated that adipocyte progenitors lose adipogenic differentiation potential that may affect lipid storage capacity of adipose tissue and lead to ectopic lipid accumulation [7, 8]. Moreover, adipose tissue aging is accompanied by the accumulation of senescent adipocyte progenitors [3]. On the other hand, removal of p16Ink4a^+^ senescent cells has been proven to be beneficial for delaying age-related disorders and increasing life span [9].

Expression levels of neurotrophic factors in adipose tissue are dynamically regulated during developmental period, and may control the innervation levels and functions of adipose tissue during perinatal periods and adulthood [10]. Especially, sympathetic innervation is required for the regulation of adipose tissue lipolysis, uncoupling protein 1 (UCP1)-dependent thermogenesis, and mitochondrial metabolism of adipose tissue [11, 12]. Regarding changes in neuronal input related to aging, reduction in sympathetic activity has been reported in brown adipose tissue with advanced age, which has been suggested as a mechanism of age-dependent decrease in brown adipose tissue activity [5, 8].

Brain-derived neurotrophic factor (BDNF) has been investigated as a neurotrophic factor that is widely distributed in brain and other peripheral tissues, functioning in the regulation of neuronal development, neuroprotection and synaptic plasticity [13]. For example, an elegant study using BDNF knockout (KO) mouse model demonstrated that BDNF expression is required for survival and maintenance of peripheral sensory neurons [14]. However, follow-up studies demonstrated cell type-specific effects of BDNF, showing BDNF KO increases innervation of skin [15, 16]. Recent studies using conditional KO model of intestinal BDNF expression also exemplified that organ-derived BDNF suppresses development of innervation [17]. Somewhat contradictory role of BDNF has been explained partly by distinct roles of mature form and pro-form of BDNF. BDNF is synthesized as pre-pro-BDNF and cleaved into pro-form that can further undergo intracellular or extracellular cleavages, generating mature form and pro-domain of BDNF [13, 18]. The pro-forms of BDNF (proBDNF) can inhibit innervation, partly by inducing neuronal apoptosis through activation of p75^NTR^ and sortilin [13, 19, 20]. However, the role of BDNF expression in adipose tissue in relation to aging has not been fully investigated.

We investigated age-dependent expression patterns of BDNF in adipose tissue and demonstrated that proBDNF expression in visceral white adipose tissue increased with age. To determine the cellular source of BDNF, we isolated progenitors from adipose tissue and found the enrichment of BDNF expression in PDGFRα^+^ adipocyte progenitor cells. We examined the correlation between BDNF expression in visceral white adipose tissue and the aging-related phenotype. The physiological role of BDNF in adipose tissue function was further studied using a PDGFRα^+^ progenitor- specific BDNF knockout mouse model (BDNF^Pdgfra ^KO).

## MATERIALS AND METHODS

### Mice

All animal protocols were approved by the Institutional Animal Care and Use Committees at Yonsei University and Seoul National University. All animal experiments were conducted in strict compliance with the guidelines for humane care and use of laboratory animals specified by the Ministry of Food and Drug Safety. Mice were housed at 22±1 °C and maintained on a 12-h light/12-h dark cycle with free access to food and water at all time. Male mice were used for the experiments. C57BL/6 mice (5-weeks old) were purchased from Central Lab. Animal Inc. For high fat diet (HFD) experiment, C57BL/6 mice were fed with diet with 60% kcal% fat (Research Diet) for 12 weeks. Pdgfra-CreER (stock#018280, B6N.Cg-Tg(PDGFRa-Cre/ERT) 467Dbe/J), Bdnf^flox/flox ^(stock #004339, Bdnftm3Jae/J)[21] mice were purchased from the Jackson Laboratory. Pdgfra-CreER mice and Bdnf^flox/flox ^mice were crossed to produce inducible adipocyte progenitor-specific BDNF KO mice (Pdgfra-CreER/Bdnf ^flox/flox^: Bdnf^Pdgfra ^KO mice). Genotyping was carried out by PCR with genomic DNA isolated from tail, as described previously [21] (Supplementary Fig. 1). For wild type control, BDNF floxed mice (WT/BDNF^flox/flox^) without CreER were used. For Cre recombination, Pdgfra-CreER/Bdnf^flox/flox^ mice and wild type (WT/Bdnf^flox/flox^) controls were treated with tamoxifen dissolved in sunflower oil (Sigma, 75 mg/kg) by oral gavage on each of 5 consecutive days. Experiments were started 10 days after the last dose of tamoxifen. For long term maintenance, 5 days of tamoxifen treatments were repeated every 2 months. For intraperitoneal glucose tolerance test, mice were given D-glucose (2?mg/ml, sigma) by intraperitoneal injection and blood glucose levels were measured at indicated time points. Energy expenditure was measured using indirect calorimetry system (PhenoMaster, TSE system, Bad Homburg, Germany), as described previously [22] Oxygen consumption rates of adipose tissue were measured by Seahorse XF24 Analyzers using XF24 Islet Capture Microplate Screen as described previously [22, 23].

For telomere length analysis, DNA was extracted from eWAT, using AccuPrep Genomic DNA Extraction Kit (Bioneer) and used for determination of telomere copy number by real-time PCR, as described previously [23]. Subcellular fractionation was performed as described previously [24]. Brie?y, from adipose tissue homogenates in fractionation buffer (containing 3 mM HEPES (pH7.4), 210 mM mannitol, 70 mM sucrose and 0.2 mM EDTA), cells and debris pellets were removed after centrifugation at 500×g for 10 min. After centrifugation of the supernatant at 10,000 × g for 10 min, pellets containing mitochondria were collected and supernatant containing non-mitochondrial fraction was centrifuged at 95000 × g for 2 h at 4 °C to obtain the plasma membrane fraction (pellet) and cytosolic fraction (supernatants).

### Western blot and gene expression analysis

Western blot analysis was performed as described previously [24]. Briefly, protein was extracted in RIPA buffer (Thermo Fisher) containing protease (Roche) and phosphatase (Thermo Fisher) inhibitors. Resolved proteins were transferred to polyvinylidene difluoride (PVDF) membranes. The membranes were incubated with blocking buffer (5% skim milk or BSA in TBST), primary and secondary antibodies. The following primary antibodies were used for western blot analysis: anti-pro-BDNF (mouse, Santa Cruz Biotechnology), anti-tyrosine hydroxylase (mouse, Merck Millipore), phospho-HSL(Ser660, rabbit, Cell Signaling), HSL (rabbit, Cell Signaling), anti-PDGFRα (goat, R&D system), PLIN1 (rabbit, Abcam), anti-p21 (rabbit, Cell Signaling), anti-TNFα (goat, R&D system), anti-Sortilin (goat, R&D system), anti-cleaved caspase 3(rabbit, Cell Signaling), anti-caspase 3(rabbit, Cell Signaling), anti-RIP3(rabbit, Cell Signaling), anti-phospho-RIP3(Thr231/Ser232, rabbit, Cell Signaling), anti-Na/K ATPase (rabbit, Abcam) Total OXPHOS Rodent WB Antibody Cocktail (Abcam), anti-βActin (mouse, Santa Cruz Bio-technology), and anti-α/βTubulin (rabbit, Cell Signaling). Quantitative PCR was performed as described previously [24]. Briefly, RNA was extracted using TRIzol® reagent (Invitrogen), and was reverse transcribed using a cDNA synthesis kit (Applied Biosystems). One hundred nanograms of cDNA was subjected to quantitative polymerase chain reaction (qPCR) in 20-μl reaction volumes (iQ SYBR Green Supermix; Bio-Rad) with 100 nM primers. qRT-PCR was performed using SYBR Green dye and CFX Connect Real-time system (Bio-Rad) for 45 cycles and fold change for all samples was calculated by using the 2-ΔΔCt method. Peptidylprolyl Isomerase A (PPIA) was used as a housekeeping gene for mRNA expression analysis. Primers used for qRT-PCR were described previously [25].

### Histology

Adipose tissue was processed for histological sections, and 5 μm-thick paraffin sections were subjected to immunohistochemical analysis, as previously described [26]. Anti-F4/80 antibody (rat, Serotech), anti-pro-BDNF (mouse, Santa Cruz Biotechnology) anti-PDGFRα (goat, R&D system), PLIN1 (rabbit, Abcam), and anti-tyrosine hydroxylase (mouse, Merck Millipore) were used. TUNEL Assay Kit-HRP-DAB (Abcam) was used to detect apoptotic cells in paraffin sections of eWAT.

### Stromovascular cells and adipocyte fractionation and MACS-isolation

Stromovascular cells (SVC) and adipocytes from eWAT were fractionated, as previously described [21, 26]. For gene expression analysis, dissociated adipose tissue was fractionated by magnetic cell sorting (MACS) with anti-PDGFRα-PE/anti-PE-microbeads, anti-F4/80-FITC/anti-FITC-microbeads, and anti-CD31-APC/anti-APC-microbeads (Miltenyi Biotech). For adipogenic differentiation, MACS-isolated PDGFRα was expanded in Dulbecco’s Modified Eagle’s Medium (DMEM) containing 10% FBS, and then differentiated by DMEM supplemented with a standard adipogenic cocktail for 7 days, as described previously [25]. To determine levels of adipogenic differentiation, differentiated cells were labeled with 4,4-difluoro-5-(2-thienyl)-4-bora-3a,4a-diaza-s-indacene-3-dodecanoic acid (BODIPY 558/568 C12) (Invitrogen Molecular Probes) or Oil Red O (Sigma-Aldrich). Mitochondrion-labeling in live cells was performed using red-?uorescent mitochondrion-selective probe MitoTracker Red CMXRos (Thermo Fisher, Waltham, MA, USA).

### Cell Cultures

The C3H10T1/2 cells (ATCC (Manassas, VA, USA)) were cultured, as previously described [22]. Brie?y, the cells were cultured in growth medium (Dulbecco’s modified Eagle’s medium (DMEM: Sigma) supplemented with 10% fetal bovine serum (FBS, Gibco Thermo Fisher Scientific, Waltham, MA, USA) and 1% penicillin/ streptomycin (Thermo Fisher, Waltham, MA, USA), and then exposed to adipogenic differentiation medium (DMEM supplemented with 10% FBS, BMP4 (20 ng/mL, R&D system, Minneapolis, MN, USA), indomethacin (0.125 mM, Cayman, Ann Arbor, MI, USA), isobutylmethylxanthine (2.5 mM, IBMX, Cayman), dexamethasone (1 µM, Cayman, Ann Arbor, MI, USA), insulin (10 µg/mL, Sigma, St. Louis, MO, USA) and triiodothyronine (T3, 1 nM, Cayman, Ann Arbor, MI, USA) for 3 days. For the maintenance of adipogenic differentiation, the cells were exposed to DMEM containing 10% FBS, 10 µg/mL insulin (Sigma, St. Louis, MO, USA) and 1 nM triiodothyronine (T3, Cayman, Ann Arbor, MI, USA) for 3 days. Fully differentiated adipocytes were exposed to DMEM supplemented with 10% FBS overnight and then treated with pro-BDNF (10ng/ml, Alomone Labs) [27, 28]. Annexin V assay was performed for detection of apoptosis by using FITC Annexin V Apoptosis Detection Kit with PI (Biolegend), according to the manufacturer’s instruction. For Sortilin knockdown, siRNA targeting Sortilin (Bioneer) was transfected into adipocytes differentiated from C3H10T1/2 cells, using Lipofectamin2000 (Thermo Fisher, Waltham, MA, USA). For cell surface protein isolation, Pierce Cell Surface Protein Isolation Kit (Thermo) was used according to manufacturer’s instruction.

**Figure 1.**
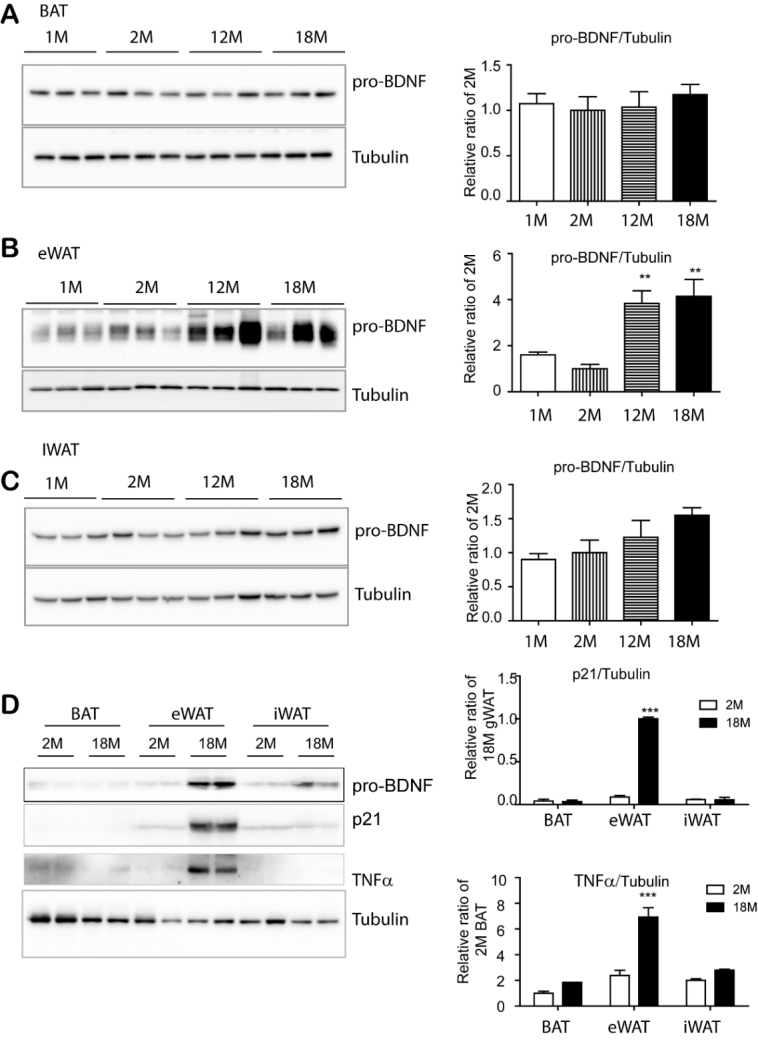
BDNF expression was upregulated in epididymal adipose tissue with advanced age, but not in brown adipose tissue and inguinal white adipose tissue. (A-D) Immunoblot analysis of BDNF and senescence marker expression in supra-scapular brown adipose tissue (BAT), epididymal white adipose tissue (eWAT), and inguinal white adipose tissue (iWAT) of mice at the indicated ages. N= 4, mean ± S.E.M, p value was calculated by t-test (Full length blots in supplementary Fig. 2).

**Figure 2.**
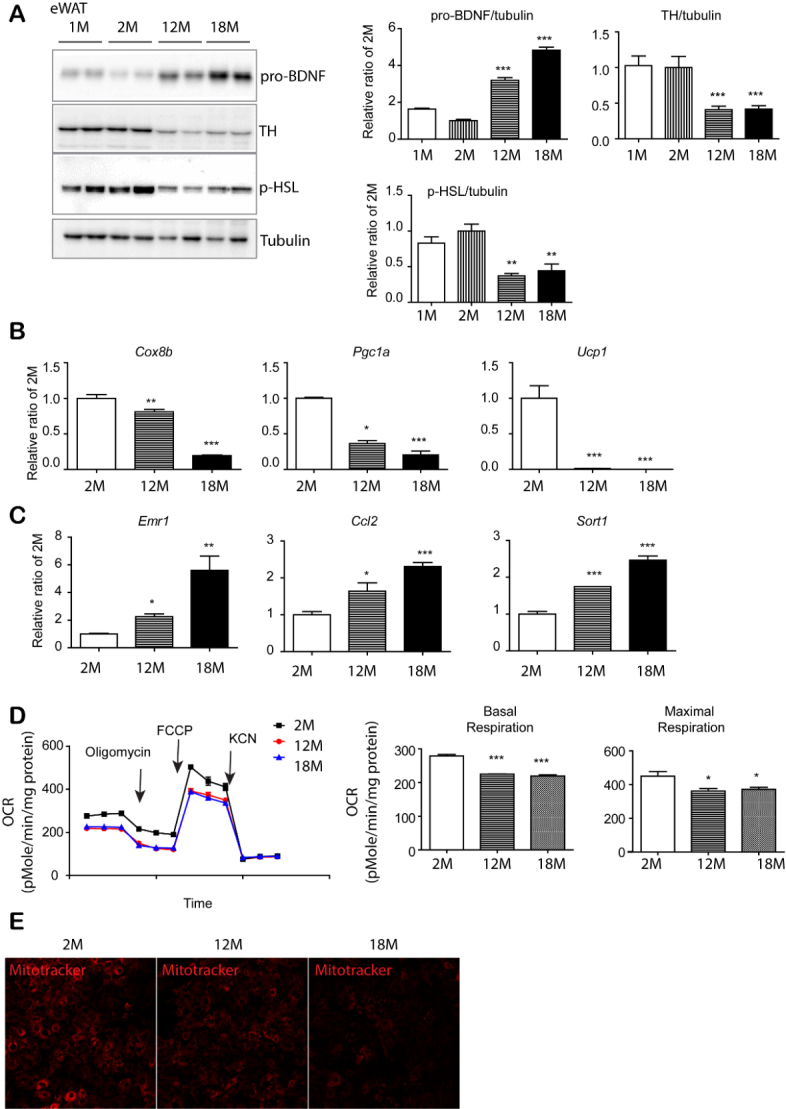
Age-dependent increase in pro-BDNF expression was associated with reduction in sympathetic innervation and mitochondrial activity in eWAT. (A) Immunoblot analysis of BDNF, TH and p-HSL expression in epididymal white adipose tissue (eWAT) of mice at the indicated ages. (n = 4, mean ± S.E.M, **p<0.01, ***p<0.001). (B, C) quantitative PCR analysis. (n = 4, mean ± S.E.M, *p<0.05, **p<0.01, ***p<0.001). (D) Analysis of oxygen consumption rate (OCR) of eWAT obtained from 2, 12 and 18-month old mice with a series of treatments of indicated drugs (oligomycin, carbonyl cyanide-4(trifluoromethoxy)phenylhydrazone (FCCP), and potassium cyanide (KCN)) (n = 3, mean ± S.E.M, *p<0.05, **p<0.01, ***p<0.001). (E) MitoTracker Red CMXRos staining in adipocytes differentiated from PDGFRα^+^ cells of eWAT of 2, 12, and 18-month-old mice.

### Statistical analysis

Statistical analyses were performed using GraphPad Prism 5 software (GraphPad Software, La Jolla, CA, USA.). Data are presented as mean ± standard errors of the means (SEMs). Statistical significance between two groups were determined by unpaired t-test. Comparisons among multiple groups was performed using a one-way or two-way analysis of variance (ANOVA), with Bonferroni post hoc tests to determine p values.

## RESULTS

### Pro-BDNF expression increased in visceral white adipose tissue with advanced age

To determine whether BDNF expression levels are altered during aging, supra-scapular brown adipose tissue (BAT), subcutaneous inguinal white adipose tissue (iWAT), and epididymal white adipose tissue (eWAT) were analyzed at the age of 1, 2, 12 and 18 months. It has been reported that BDNF is synthesized as pre-pro-BDNF that can be cleaved into proBDNF (~32kD), which can be further processed into pro-domain of BDNF (~17kD) [29] and mature BDNF (~14kD) [30]. While mature BDNF (~14kD) was not clearly detectable in adipose tissue by immunoblotting, the major form detected in overall adipose tissue was pro-BDNF (~32kD). As shown in Figure 1, pro-BDNF expression was not significantly altered in BAT with aging, while there was a slight increase in iWAT. However, eWAT of 12 and 18-month-old mice demonstrated dramatic increase in pro-BDNF expression, which was approximately 4-fold increase as compared to the expression in 2-month-old mice. (Fig. 1B). Among adipose depots, pro-BDNF expression was highest in the eWAT of 18-month-old mice. Expression levels of senescence associated genes, such as cyclin-dependent kinase inhibitor 1(p21) and tumor necrosis factor-alpha (TNFα), were upregulated in eWAT of 18-month-old mice, compared to their younger counterparts (Fig. 1D). These data suggested that eWAT-specific increase in proBDNF expression might be involved in the aging phenotype of eWAT.

**Figure 3.**
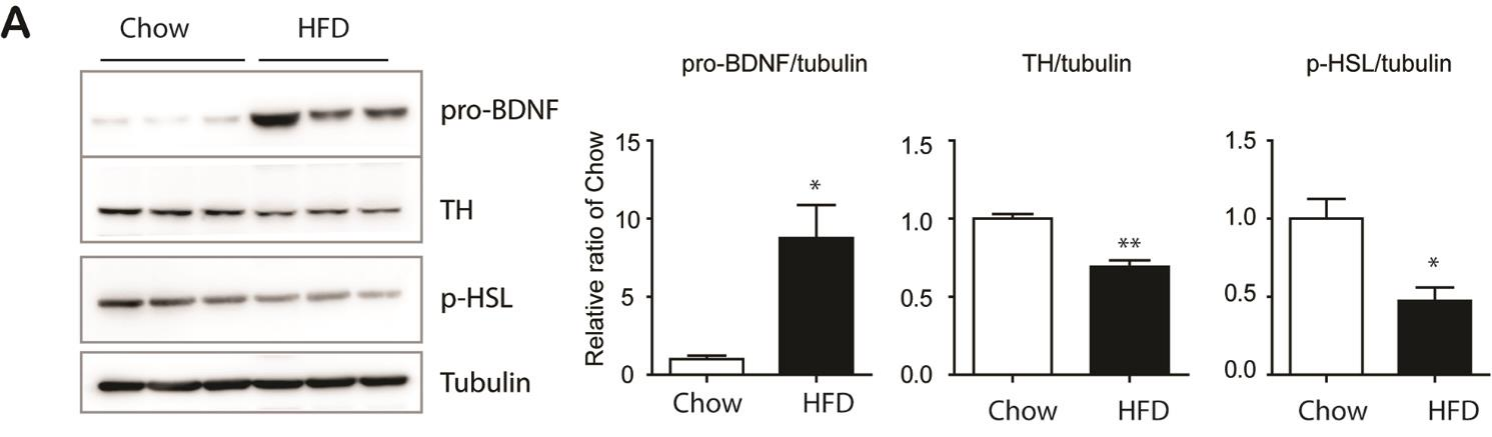
Upregulation of pro-BDNF expression by 12 weeks of HFD feeding (A) Immunoblot analysis of BDNF, TH and p-HSL expression in epididymal white adipose tissue (eWAT) mice fed with high fat diet and chow diet for 12 weeks. (n = 6, mean ± S.E.M, *p<0.05, **p<0.01).

### Increase in pro-BDNF expression in visceral white adipose tissue was associated with reduction in sympathetic innervation with aging

Next, we measured the expression levels of a rate limiting enzyme in catecholamine synthesis, tyrosine hydroxylase (TH), which can be used to assess sympathetic innervation levels [24]. Our data indicated that TH expression was downregulated, reaching 60% reduction at 12- and 18-month age compared to the expression in 2-month-old mice (Fig. 2A). Since sympathetic stimulation is related to PKA-dependent lipolysis through beta 3-adrenergic receptor, we examined the major PKA-responsive lipase, hormone sensitive lipase phosphorylation (p-HSL) levels. Results indicated that p-HSL showed reduced levels with increasing age (Fig. 2A), supporting the reduction in sympathetic innervation with advanced age. It has been reported that the levels of sympathetic innervation are related to mitochondrial activity in adipose tissue [5, 11, 24]. qPCR analysis also demonstrated that genes related to mitochondrial biogenesis and activity [cytochrome c oxidase subunit 8B (Cox8b), peroxisome proliferator-activated receptor gamma coactivator 1 alpha (Ppargc1a), and Ucp1] were downregulated with advanced age (Fig. 2B and Supplementary Fig. 4). Data also demonstrated increased expression of EGF-like module-containing mucin-like hormone receptor-like 1 (Emr1) (a gene encoding F4/80: a macrophage marker) and C-C motif chemokine ligand 2 (Ccl2) (a pro-inflammatory cytokine) with advanced age (Fig. 2C). While m-BDNF promotes cell growth and survival though activation of both BDNF/NT-3 growth factors receptor (neurotrophic tyrosine kinase receptor type 2: Ntrk2) and tumor necrosis factor receptor superfamily member 16 (p75 neurotrophin receptor), it has been reported that pro-BDNF promotes cell death through p75NTR and sortilin [20, 27], which might be involved in the reduction in sympathetic innervation with increased pro-BDNF expression. Thus, we examined pro-BDNF receptor expression and found that Sort1 expression in eWAT was significantly increased in 12 and 18-month-old mice (Fig. 2C), suggesting that sortilin induction might be associated with a reduction in sympathetic innervation. A decrease in mitochondrial metabolic activity was confirmed by the reduced oxygen consumption rate in the eWAT of 12-month and 18-month-old mice (Fig. 2D). MitoTracker staining was also reduced in adipocytes differentiated from pre-adipocytes isolated from eWAT (Fig. 2E), which further demonstrated the reduction in the mitochondrial activity of eWAT in 12- and 18-month old mice.

Since obesity has been characterized by accelerated aging of adipose tissue, we examined pro-BDNF expression levels in 12-week HFD-fed mice. As shown in Figure 3, pro-BDNF expression was upregulated in eWAT of HFD-treated group. Also, reduction in TH and p-HSL levels was observed in HFD-fed mice, suggesting that eWAT-specific upregulation of pro-BDNF expression through HFD consumption may contribute to the aging phenotype observed in the visceral adipose tissue of obese individuals.

**Figure 4.**
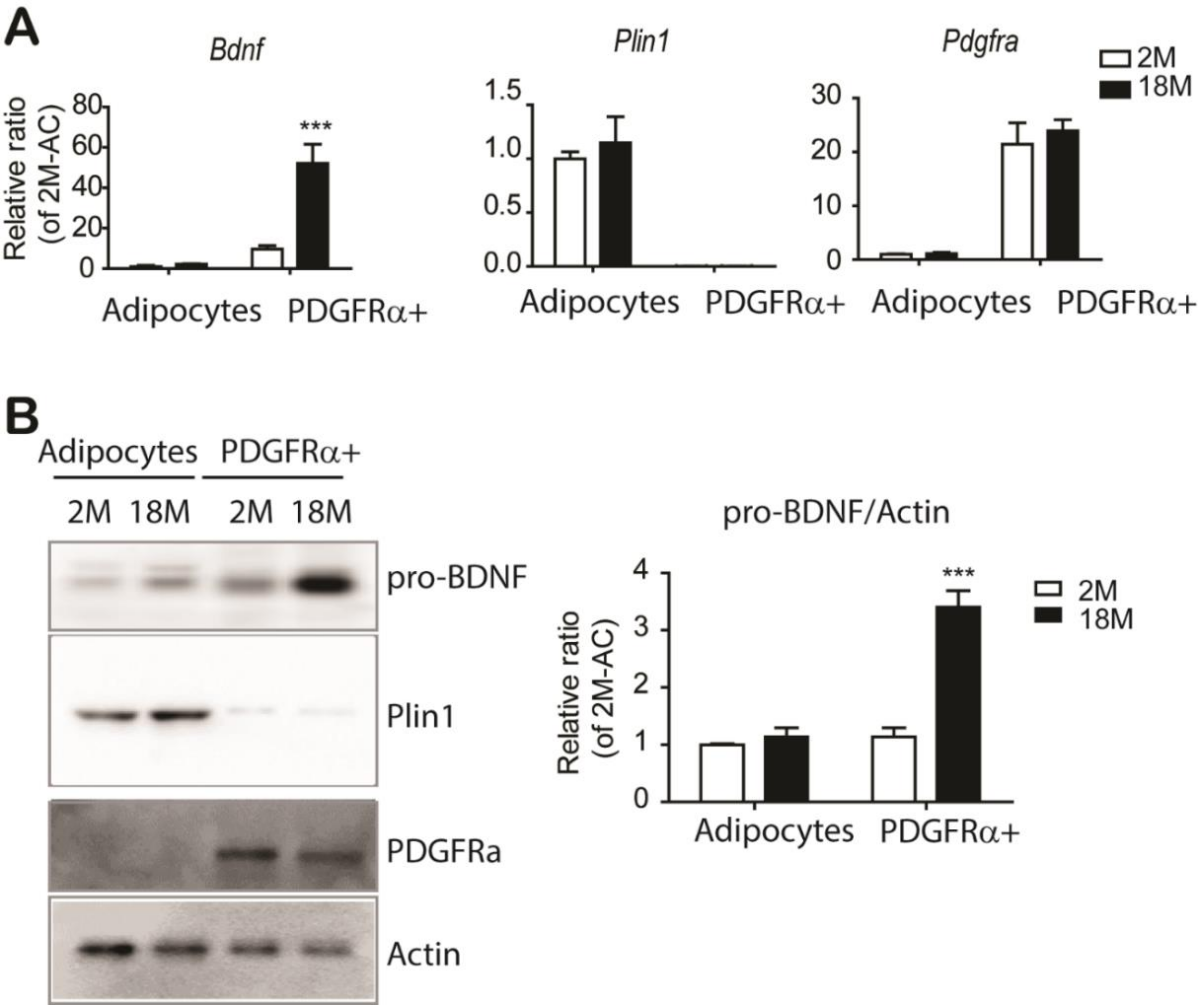
The major cellular source of pro-BDNF expression in eWAT is PDGFRα^+^ adipocyte progenitors. (A) quantitative PCR analysis of Bdnf expression in adipocytes and PDGFRA^+^ cells isolated from eWAT of mice at the indicated ages (n = 3, mean ± S.E.M, ***p<0.001). Plin1 and Pdgfra expressions were used as specific markers for adipocytes and progenitor cells, respectively. (B) Immunoblot analysis of BDNF expression in adipocytes and PDGFRA^+^ cells isolated from eWAT of mice at the indicated ages (n = 3, mean ± S.E.M, ***p<0.001). Full images of Western blots are shown in supplementary Fig. 4.

### Pro-BDNF expression upregulated in PDGFRA^+^ cells with advanced age

To determine the cellular source of BDNF expression in adipose tissue, we fractionated PDGFRA^+^ adipocyte progenitor cells and adipocytes [27] and examined cell type-specific gene and protein expression. As expected, expression of an adipocyte specific maker, perilipin 1 (Plin1) was restricted to adipocyte fractions (Fig. 4A and 4B). Compared to adipocyte fraction, BDNF expression was enriched in PDGFRA^+^ adipocyte progenitors (Fig. 4A). Consistent with qPCR analysis, immunoblot analysis confirmed the enrichment of pro-BDNF in PDGFRA^+^ cells (Fig. 4B). PDGFRA^+^ adipocyte progenitors showed upregulation of pro-BDNF expression with advanced age (Fig. 4B). In addition, we examined pro-BDNF expression in stromovascular fractions, including F4/80^+^ macrophages and CD31^+^ endothelial cells, and found that pro-BDNF expression was enriched in PDGFRA^+^ cells. (Supplementary Fig. 9). Immunohistochemical analysis also indicated robust expression of pro-BDNF in PDGFRA^+^ cells (Supplementary Fig. 10).

**Figure 5.**
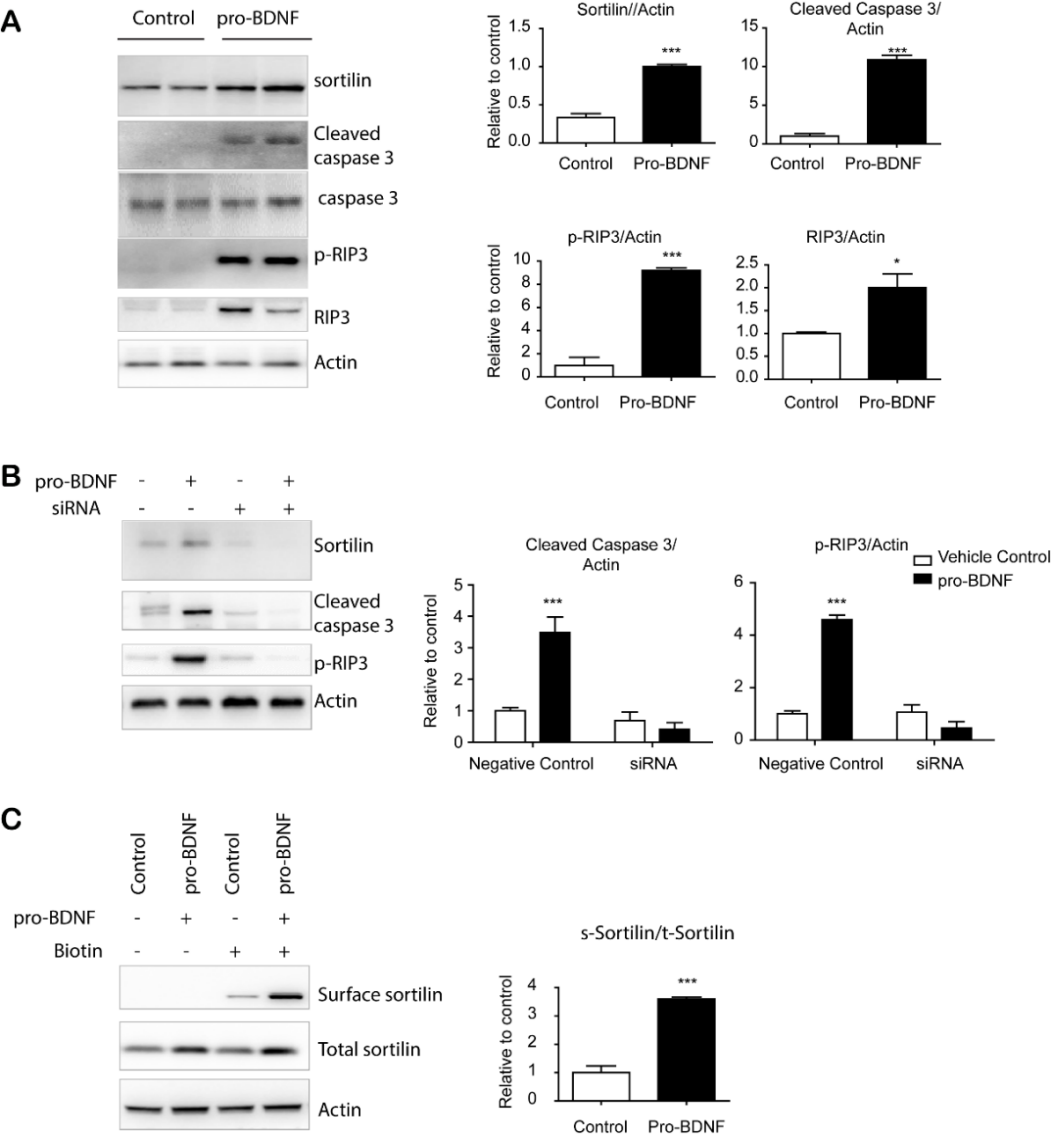
proBDNF treatment-induced apoptosis/necroptosis of adipocytes required sortilin expression. (A) Immunoblot analysis of sortilin expression and apoptosis/necroptosis markers in adipocytes differentiated from C3H10T1/2 cells. (B) Immunoblot analysis of sortilin in adipocytes differentiated from C3H10T1/2 treated with siRNA or scrambled sequence controls (negative controls) (mean ± SEM; n = 4, *** p < 0.001). (C) Immunoblot analysis of cell surface protein detection in adipocytes differentiated from C3H10T1/2 cells treated with vehicle or pro-BDNF (10ng/ml) for 24 h (n = 4, means ± SEM, *** p < 0.001). Full images of Western blots are shown in supplementary Fig. 5.

**Figure 6.**
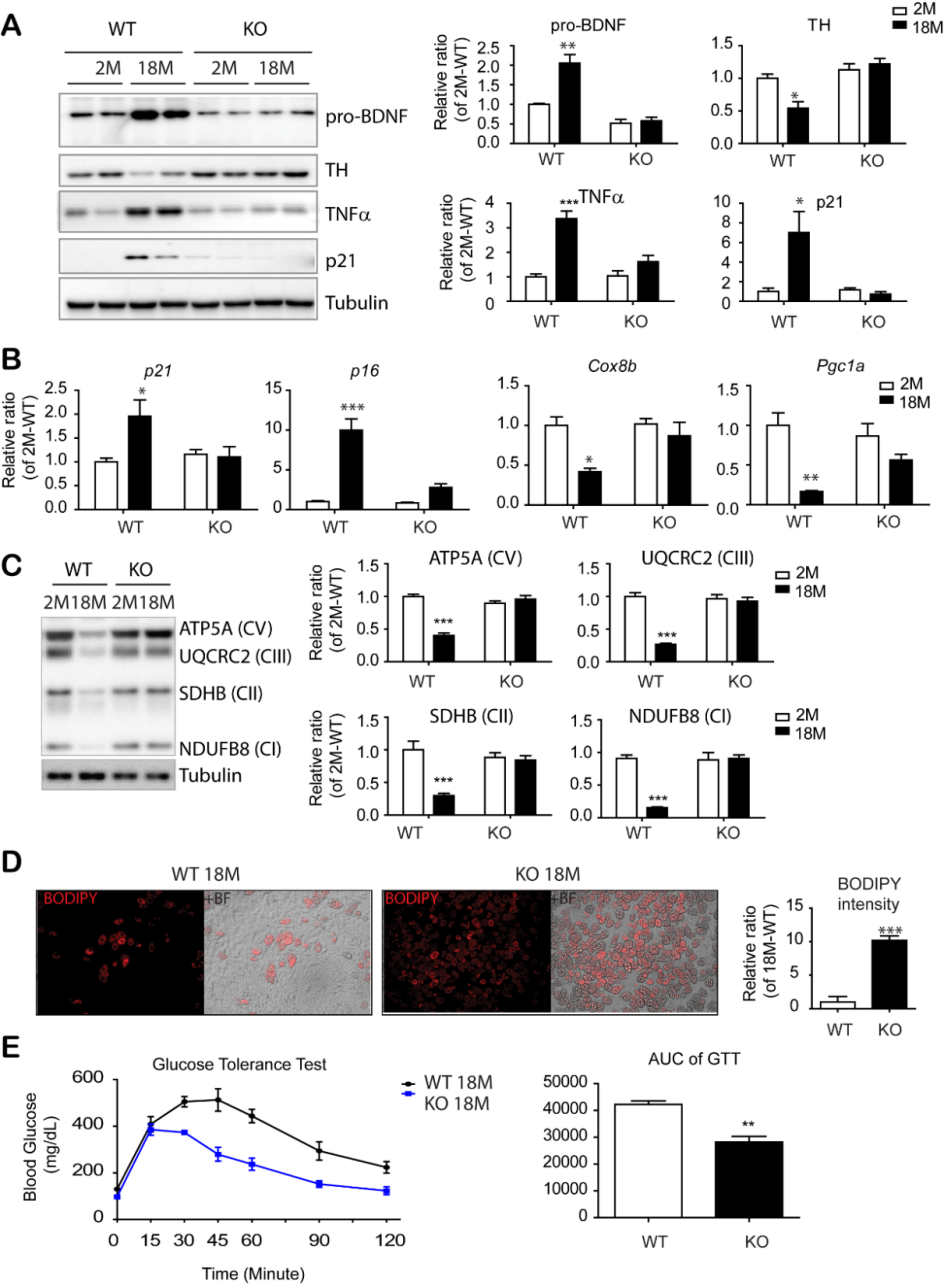
PDGFRα^+^ cell-specific KO reduced inflammatory and senescence marker expression in eWAT and insulin resistance of mice with advanced age. (A) Immunoblot analysis of BDNF and TH expression in epididymal white adipose tissue (eWAT) of BDNF^pdgfra ^KO and WT mice at the indicated ages. (n = 5 per condition, mean ± S.E.M, *p<0.05, **p<0.01, *** p < 0.001). (B) Quantitative PCR analysis of eWAT of BDNF^pdgfra ^KO and WT mice at the indicated ages. (n = 5, mean ± S.E.M, *p<0.05, **p<0.01, ***p<0.001). (C) Immunoblot analysis of mitochondrial makers involved in mitochondrial oxidative phosphorylation. (D) BODIPY staining of adipocytes differentiated from PDGFRα^+^ cells that were isolated from eWAT of BDNF^pdgfra ^KO and WT mice (n = 4, mean ± S.E.M, **p<0.01). (E) Measurement of glucose tolerance test (GTT) in WT and BDNF^pdgfra^KO mice and the area under the curve of GTT plots. N = 5, mean ± S.E.M, **p<0.01. Full images of Western blots are shown in supplementary Fig. 6.

### proBDNF-induced apoptosis/necroptosis in adipocytes required sortilin expression

To investigate the roles of pro-BDNF in adipose tissue dysfunction, we treated adipocytes differentiated from C3H10T1/3 cells with pro-BDNF. Interestingly, pro-BDNF treatment increased sortilin protein levels as well as markers of apoptosis (cleaved caspase 3) and necroptosis [phosphor-receptor-interacting protein kinase 3 (RIP3)) (Fig. 5A). Flow cytometric detection of Annexin V further demonstrated the induction of apoptosis by pro-BDNF treatment (Supplementary Fig. 11). siRNA knockdown of sortilin expression in differentiated adipocytes prevented proBDNF-induced apoptosis, supporting the importance of sortilin expression in proBDNF-mediated signaling during adipose tissue aging (Fig. 5B). Next, we examined the cell surface localization of sortilin as a receptor and found its localization in the plasma membrane was increased by proBDNF treatment (Fig. 5C).

**Figure 7.**
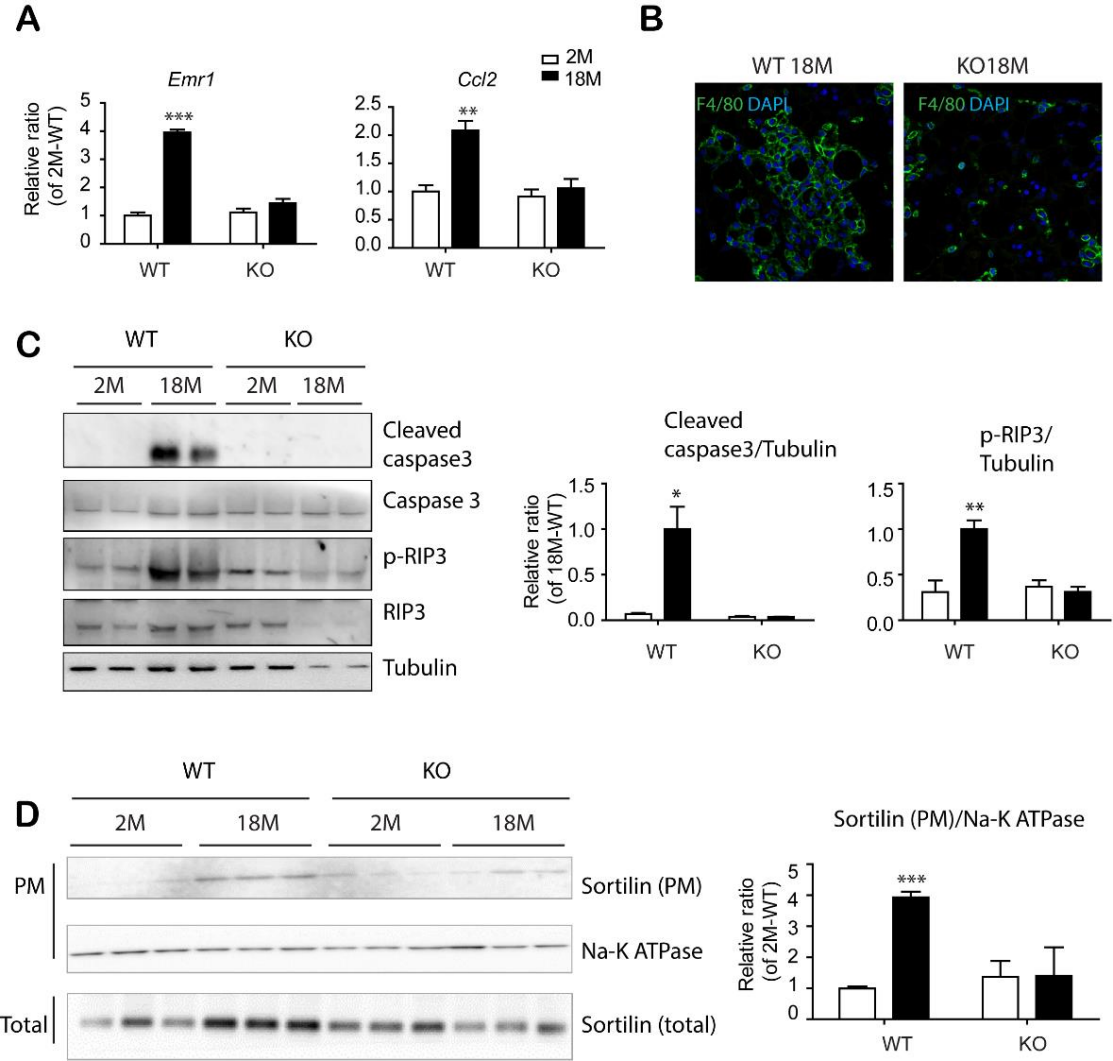
PDGFRα^+^ cell-specific KO reduced apoptosis and necroptosis in eWAT of mice with advanced age. (A) Quantitative PCR analysis of eWAT of BDNF^pdgfra ^KO and WT mice at the indicated ages. (n = 5, mean ± S.E.M, *p<0.05, **p<0.01, ***p<0.001). (B) Immunostaining of F4/80 in paraffin sections of eWAT of BDNF^pdgfra ^KO and WT mice. DAPI was used as a nuclear counterstain. (C) Immunoblot analysis of apoptosis/necroptosis makers in eWAT of BDNF^pdgfra ^KO and WT mice. (D) Immunoblot analysis of sortilin expression in plasma membrane fractions of eWAT of WT and BDNF^pdgfra ^KO mice (n = 5, means ± SEM). Full images of Western blots are shown in supplementary Fig. 7.

### PDGFRA^+^ cell-specific BDNF knockout prevented aging-related phenotypes in eWAT

To further study the in vivo physiological function of BDNF in adipose tissue, we established PDGFRA^+^ cell- specific knockout of BDNF (BDNF^Pdgfra ^KO mice). Additionally, we used tamoxifen-inducible Cre/Lox system to avoid potential developmental effect of BDNF deletion. Upregulation of pro-BDNF expression in eWAT during aging was prevented by PDGFRA^+^ cell-specific BDNF knockout (Fig. 6A). In addition, the age-related reduction in TH expression was not observed in BDNF^Pdgfra ^KO mice (Fig. 6A and S12). Moreover, immunoblot analysis and qPCR analysis demonstrated that aging-related reduction in mitochondrial marker expression and increase in senescence marker expression (i.e. p16, p21 and TNFα) in eWAT was attenuated in PDGFRA^+^ cell-specific BDNF KO mice (Fig. 6A, 6B and 6C). Furthermore, the reduction in telomere length by aging was attenuated in BDNF^Pdgfra^ KO mice (Supplementary Fig. 13).

Next, PDGFRA^+^ adipocyte progenitors were isolated from eWAT of 18-month-old mice by MACS and cultured using standard adipogenic differentiation media. BODIPY and Oil Red O staining indicated that progenitors from eWAT of BDNF^Pdgfra^ KO mice possessed higher adipogenic differentiation potential than wild type controls (Fig. 6D and S14). Although there was no significant difference in basal energy expenditure between WT and BDNF^Pdgfra^KO mice (Supplementary Fig. 15), intraperitoneal glucose tolerance tests demonstrated that aging-related insulin resistance was prevented by PDGFRA^+^ cell-specific BDNF KO (Fig. 6E). Furthermore, BDNF^Pdgfra ^KO mice showed reduced expression of inflammatory markers and infiltration of F4/80^+^ cells in eWAT of BDNF^Pdgfra ^KO mice, compared to wild type control conditions (Fig. 7A, 7B and supplementary Fig. 16). Reduction in inflammatory markers were correlated with reduction in expression levels of apoptosis/necroptosis markers (Fig. 7C and supplementary Fig. 17) and cell surface localization of sortilin in eWAT of 18-month-old BDNF KO mice, compared to WT counterparts (Fig. 7D). Collectively, these results suggested that inhibition of BDNF expression in adipocyte progenitors is potentially beneficial to prevent age-related adipose tissue dysfunction.

### DISCUSSION

Current study identified age-dependent increase of pro-BDNF expression in PDGFRα^+^ adipocyte progenitors and focused on the role of progenitor-derived BDNF in adipose tissue aging. Our data indicated that progenitor-derived pro-BDNF expression negatively regulated the sympathetic innervation and consequently affected metabolic and immune function of visceral adipose tissue of aged mice.

Two major mechanisms of action of BDNF on energy metabolism have been described as: 1) effect thorough central nervous system such as regulation of feeding behaviors, and 2) direct effect on peripheral tissue [31]. Overall, treatment with recombinant BDNF was beneficial to treat obesity and diabetes in rodent models [32-34]. The findings from the current work would add an important aspect of adipose tissue-derived pro-BDNF expression, playing a key role in adipose tissue aging. Furthermore, our data suggested that progenitor-specific reduction in pro-BDNF expression would be a promising strategy to prevent and treat chronic disorders related to adipose tissue aging, such as type II diabetes. Although we have demonstrated beneficial effects of PDGFRA^+^ cell-specific BDNF knockout, the current study did not examine the effects of overexpression of pro-BDNF in adipose tissue dysfunction in vivo. Further studies of pro-BDNF introduction in eWAT by genetic engineering would be required to improve our understanding of pathophysiologic roles of pro-BDNF expression.

Several studies have investigated the roles of BDNF in aging-associated dysfunction, especially in relation to neurological disorders[35]. For instance, enhancement of BDNF expression in the hippocampus has been a promising strategy to treat Alzheimer’s disease in mouse models[36]. However, studies of age-related pro-BDNF expression in various organs are currently lacking. Future investigations are needed to characterize tissue-specific roles of pro-BDNF expression in aging-related disease models.

Sortilin belongs to a family of vacuolar protein sorting 10 protein (VPS10P)-domain receptors that mediates vesicular trafficking [37]. Although it was originally identified in the brain, Sortilin 1 is expressed in other metabolically active tissues such as the liver, adipose tissue and muscle [37]. It functions as a receptor of proBDNF and typically induces neuronal apoptosis during development, pathological conditions and aging [37]. Consistent with its roles in neuronal viability, the current study demonstrated that sortilin expression in adipocytes was required for an apoptotic response to pro-BDNF. Previous studies have reported that sortilin deficiency prevents metabolic dysfunction caused by diet induced obesity [38]. Additionally, sortilin has been suggested to be involved in the regulation of Glut4 trafficking and the insulin sensitivity of adipocytes. In the present study, we speculated that an aging-induced increase in the plasma membrane localization of sortilin might impair retrograde transport of Glut4 and consequently result in dysregulation of the insulin responsive Glut4 compartment in adipocytes.

One of interesting findings from this study was the identification of PDGFRα^+^ progenitors as the cellular sources of the increased pro-BDNF expression in adipose tissue with increasing age. Thus, this study focused on the effect of progenitor-derived BDNF expression on adipose tissue aging. This finding might be related to the loss of adipogenic potential of adipocyte progenitors in aged adipose tissue [7]. Although it is not investigated in this study, it would be informative to study the effect of pro-BDNF expression on the alteration of progenitor proliferation and differentiation potential and the underlying mechanisms. Furthermore, it would be interesting to investigate the roles of BDNF in other organs in the context of stem cell aging.

The expression of pro-BDNF in adipose tissue was specific to visceral adipose tissue, eWAT. Thus, the current work suggested that pro-BDNF may lead to visceral adipose tissue-specific dysfunction with aging. Adipose tissue can be found in various anatomical locations and its function varies depending on the depots [7]. For example, subcutaneous adipose tissue possesses higher potential to be converted into catabolic brown/beige adipose tissue upon thermogenic stimuli [26]. In addition, insulin sensitivity of subcutaneous adipose tissue contributes to metabolic health by preventing ectopic lipid accumulation and subsequent lipotoxicity [7]. On the other hand, increased mass in visceral adipose tissue represents a risk factor for metabolic syndrome [1]. Likewise, changes in fat distribution with aging correspond to the pattern observed in metabolically unhealthy obesity, namely, increased ratio of visceral to subcutaneous adipose tissue [11]. It is important for future study to address the role of BDNF expression in adipose tissue distribution and depot-specific effect of progenitor derived-BDNF expression.

Collectively, these results identified upregulation of pro-BDNF expression in adipocyte progenitors as a feature of adipose tissue aging and suggested that inhibition of BDNF expression in adipocyte progenitors is potentially beneficial to prevent age-related adipose tissue dysfunction.

## Supplementary Materials

The Supplemenantry data can be found online at: www.aginganddisease.org/EN/10.14336/AD.2019.0810.
