# Supplementary material for: Aging-Induced Brain-Derived Neurotrophic Factor in Adipocyte Progenitors Contributes to Adipose Tissue Dysfunction
Source: Aging Dis. 2019 Aug 10;11(3):575–87. doi: 10.14336/AD.2019.0810 (PMC7220283; doi:10.14336/AD.2019.0810)
Supplement: Supplementary file 1 — The Supplemenantry data can be found online at: www.aginganddisease.org/EN/10.14336/AD.2019.0810. [file AD-11-3-575-s.pdf]

SUPPLEMENTARY DATA

Aging-Induced Brain-Derived Neurotrophic Factor in Adipocyte Progenitors Contributes to Adipose Tissue Dysfunction

Hyun-Doo Song[1,#], Sang Nam Kim[2,#], Abhirup Saha[2,#], Sang-Yeop Ahn[1], Seun Akindehin[1], Yeonho Son[2], Yoon Keun Cho[2], MinSu Kim[2], Ji-Hyun Park[2], Young-Suk Jung[3], Yun-Hee Lee[2*]

[1]College of Pharmacy, Yonsei University, Incheon, Republic of Korea. [2]College of Pharmacy and Research Institute of Pharmaceutical Sciences, Seoul National University, Seoul, Republic of Korea. [3]College of Pharmacy, Pusan National University, Busan, Republic of Korea.

© 2019. Song HD et al. Published online at http://www.aginganddisease.org/EN/10.14336/AD.2019.0810

SUPPLEMENTARY DATA

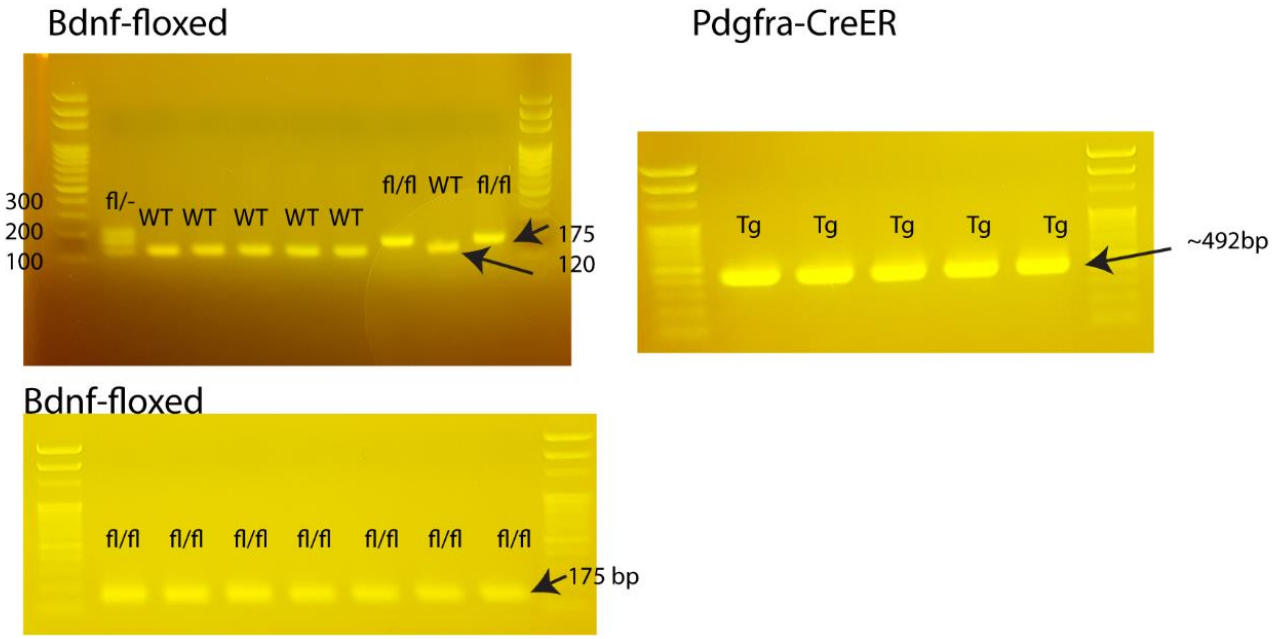

Supplementary Figure 1. Genotyping of BDNF floxed mice and Pdgfra_CreERT2 mice.

© 2019. Song HD et al. Published online at http://www.aginganddisease.org/EN/10.14336/AD.2019.0810

SUPPLEMENTARY DATA

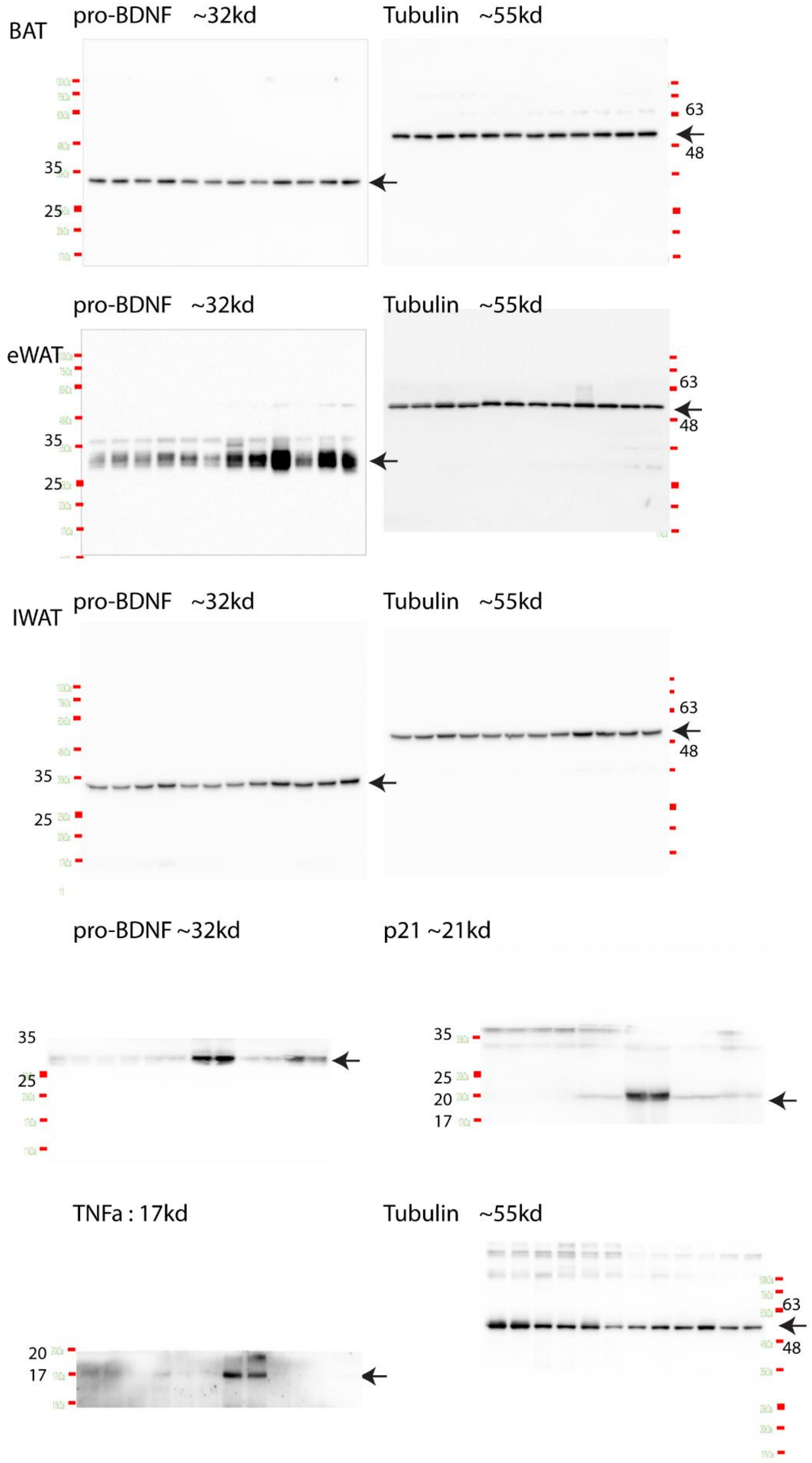

Supplementary Figure 2. Membrane images of western blot analyses used for Figure 1.

© 2019. Song HD et al. Published online at http://www.aginganddisease.org/EN/10.14336/AD.2019.0810

SUPPLEMENTARY DATA

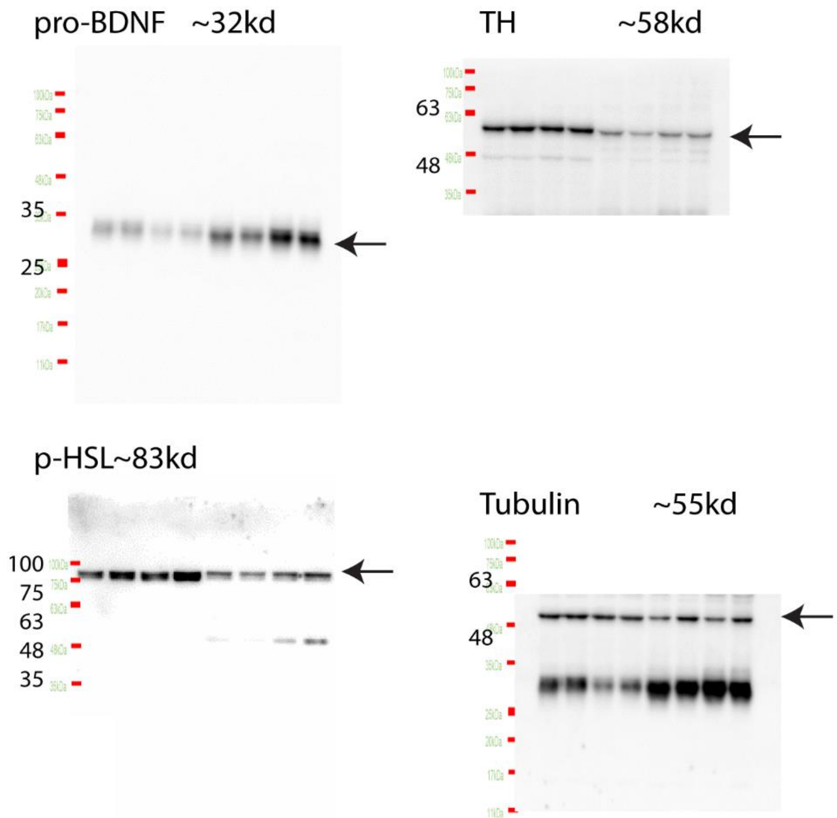

Supplementary Figure 3. Membrane images of western blot analyses used for Figure 2.

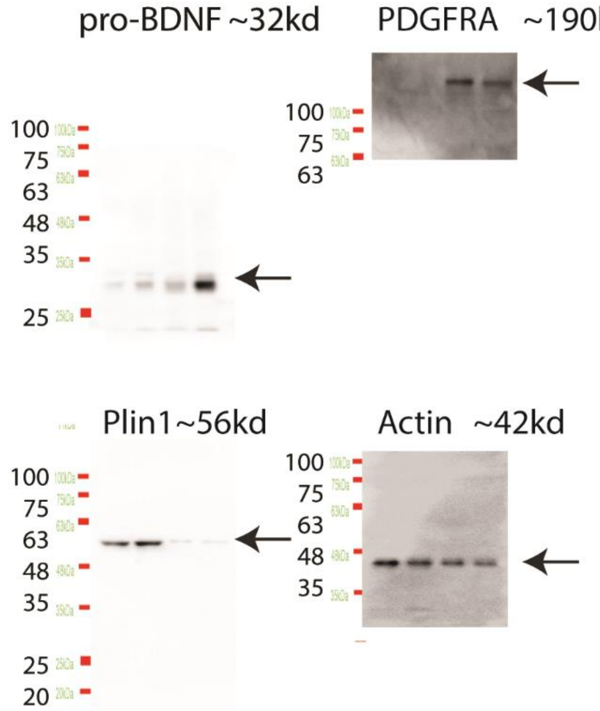

Supplementary Figure 4. Membrane images of western blot analyses used for Figure 3 and 4.

© 2019. Song HD et al. Published online at http://www.aginganddisease.org/EN/10.14336/AD.2019.0810

SUPPLEMENTARY DATA

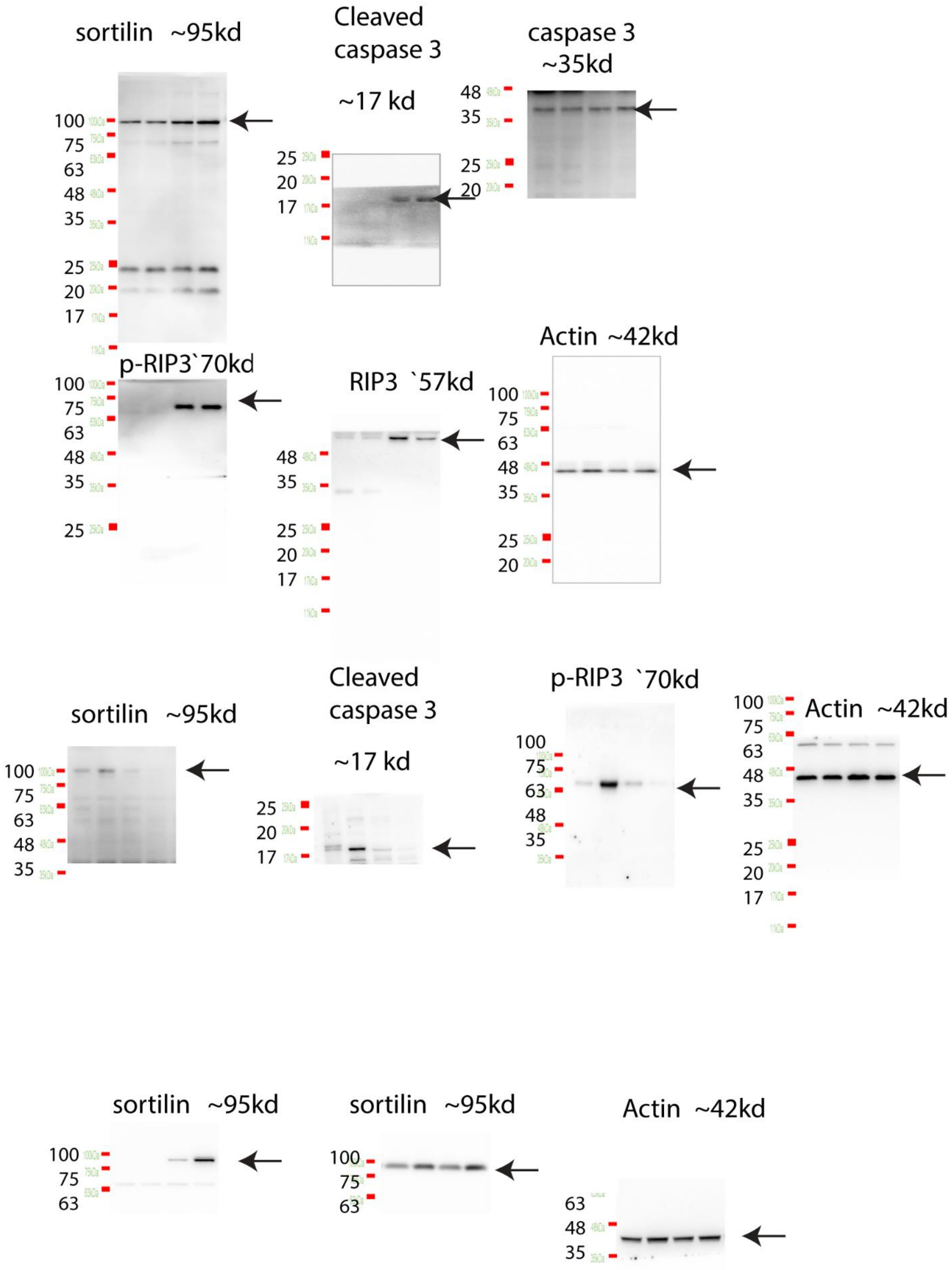

Supplementary Figure 5. Membrane images of western blot analyses used for Figure 5.

© 2019. Song HD et al. Published online at http://www.aginganddisease.org/EN/10.14336/AD.2019.0810

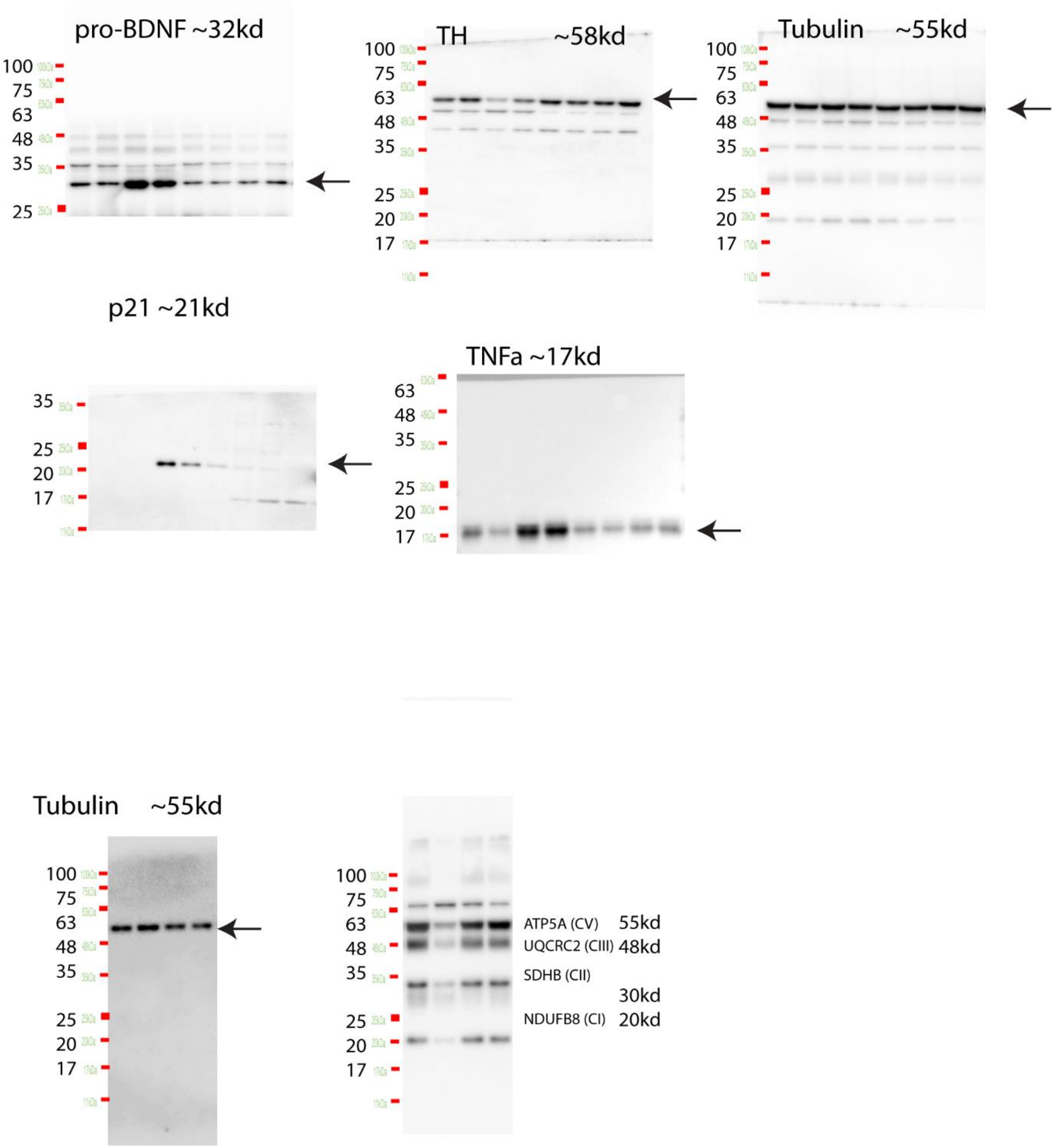

Supplementary Figure 6. Membrane images of western blot analyses used for Figure 6.

© 2019. Song HD et al. Published online at http://www.aginganddisease.org/EN/10.14336/AD.2019.0810

SUPPLEMENTARY DATA

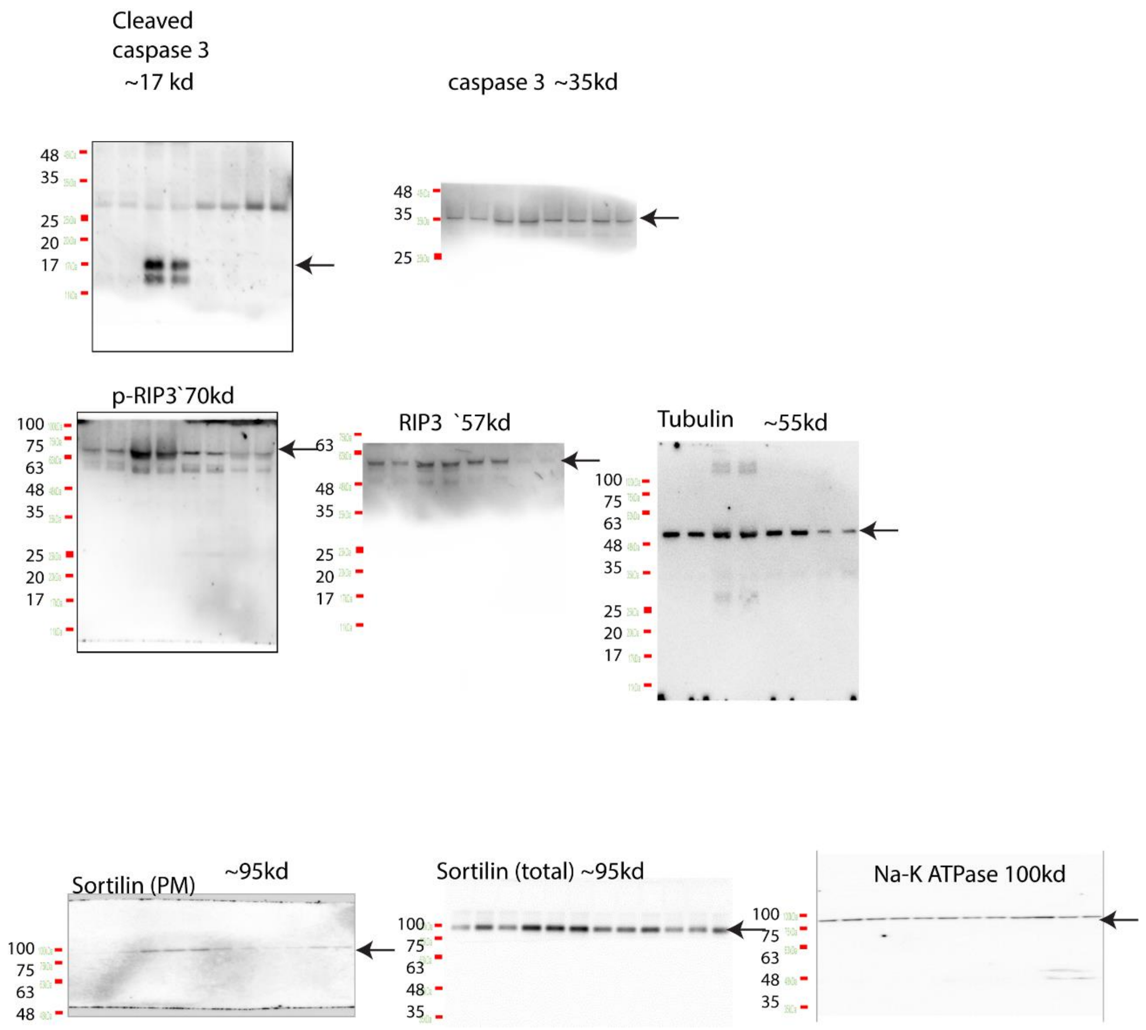

Supplementary Figure 7. Membrane images of western blot analyses used for Figure 7.

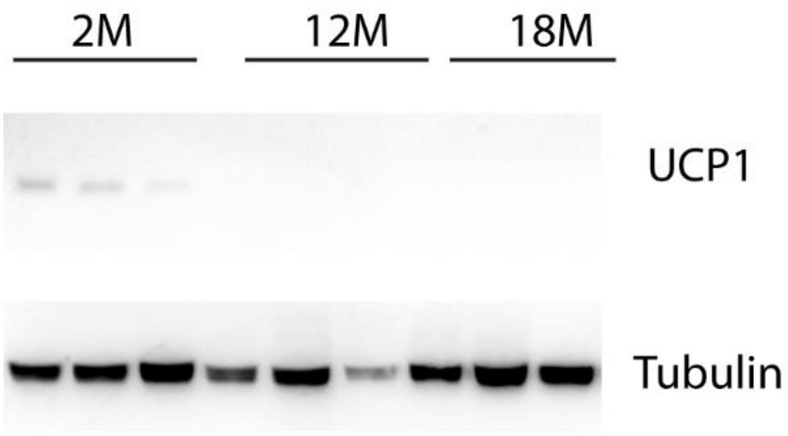

Supplementary Figure 8. Immunoblot analysis of UCP1 expression in eWAT of mice.

© 2019. Song HD et al. Published online at http://www.aginganddisease.org/EN/10.14336/AD.2019.0810

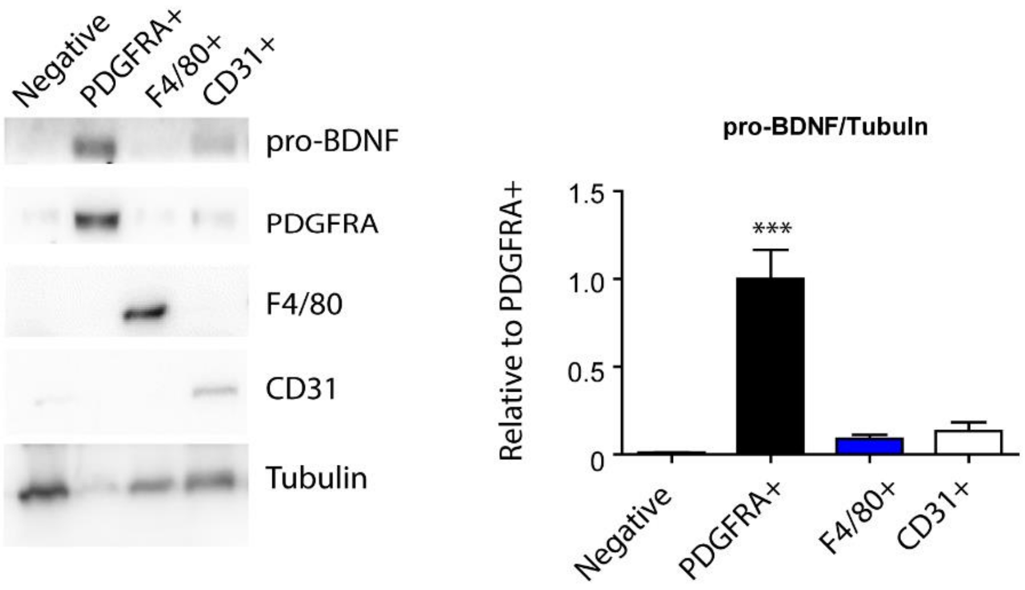

Supplementary Figure 9. Immunoblot analysis of pro-BDNF expression in stormovascular fractions of eWAT of 18-month-old WT mice. (n=3, t-test (comparison to levels in F4/80+ cells), ***p<0.001, Negative: F4/80-, CD31-, PDGFRA- stromovascular fraction).

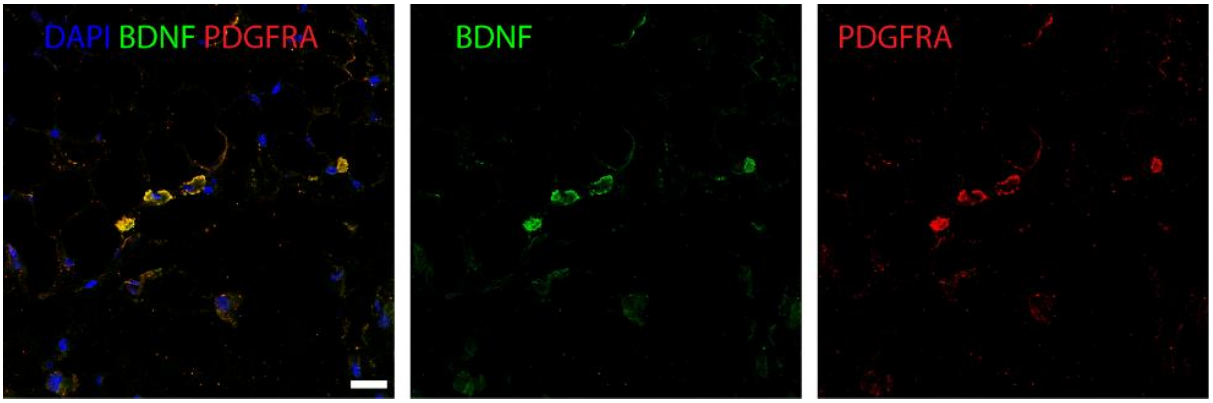

Supplementary Figure 10. Representative images of BDNF and PDGFRA+ expression in paraffin sections of eWAT of 18-month-old mice. Nuclei were counterstained with DAPI. Size bar = 20µm.

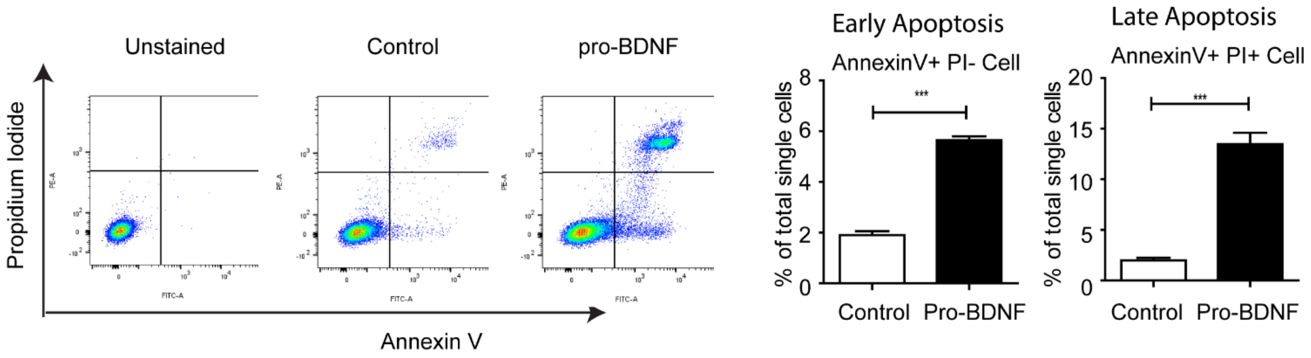

Supplementary Figure 11. Flow cytometric analysis of Annexin V/PI-stained, apoptotic cells after 24hrs of pro-BDNF treatment (10ng/ml) (n = 3, t-test, ***p<0.001).

© 2019. Song HD et al. Published online at http://www.aginganddisease.org/EN/10.14336/AD.2019.0810

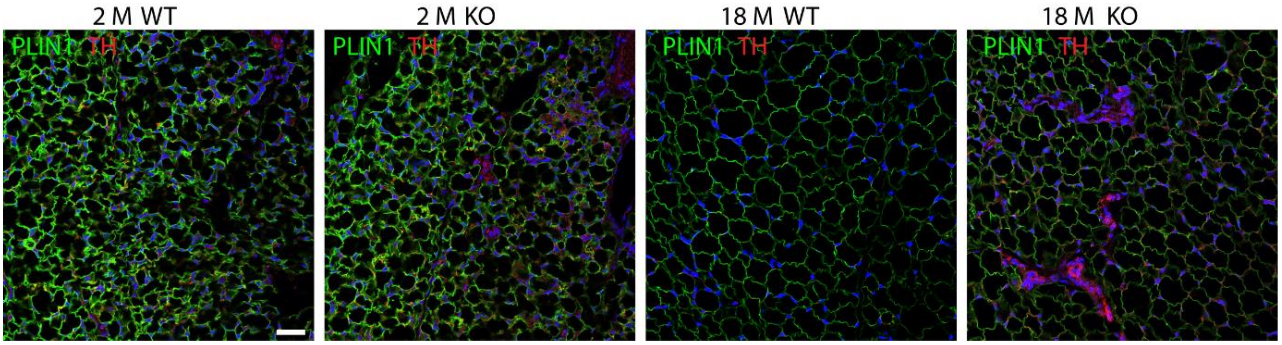

Supplementary Figure 12. Representative images of double staining for tyrosine hydroxylase (TH) and perilipin (PLIN1) in paraffin sections of eWAT of BDNF^{Pdgfra}KO and WT mice. Nuclei were counterstained with DAPI (Size bar = 100μm).

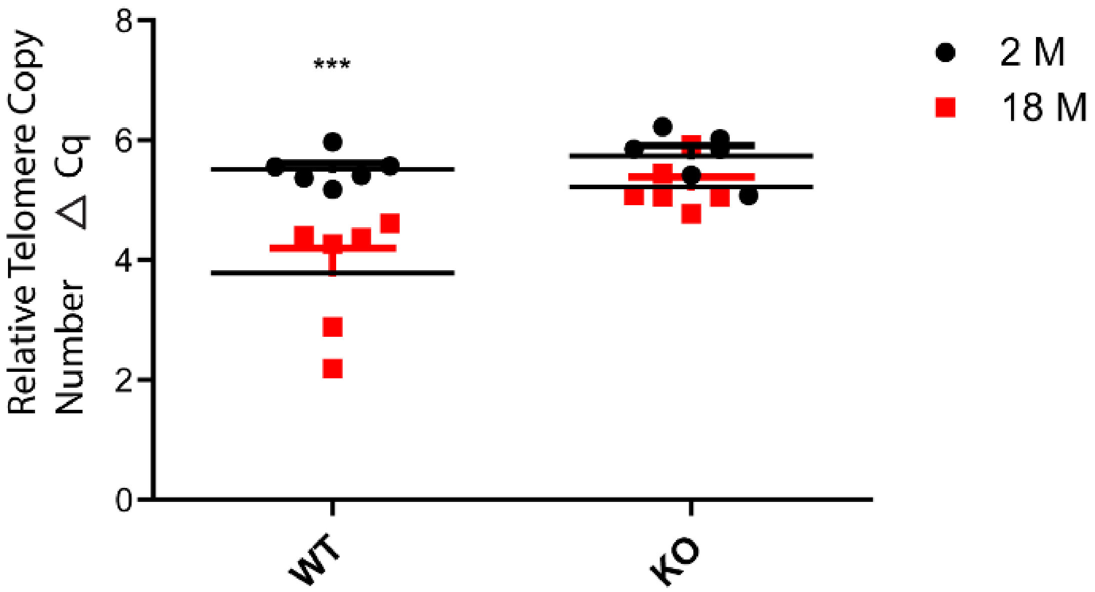

Supplementary Figure 13. Comparison of relative telomere copy number in eWAT between 2-month- and 18- month-old mice of BDNF^{Pdgfra}KO mice and WT controls. (n = 6, t-test, ***p<0.001). ***36B4*** **was used for a single copy gene control.**

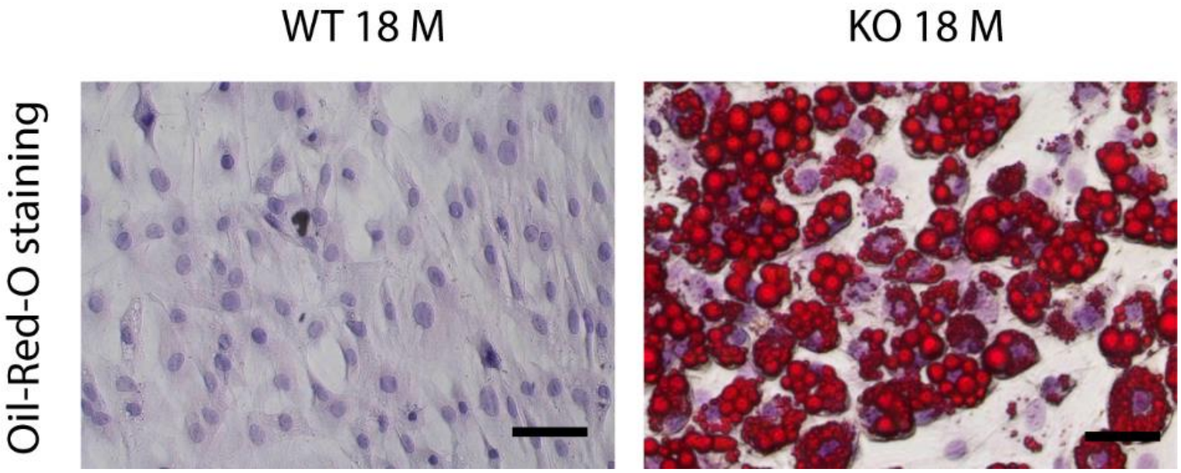

Supplementary Figure 14. Oil Red O staining of differentiated progenitor cells obtained from eWAT of BDNF^{Pdgfra} KO mice and WT control mice (size bars = 25μm). Hematoxylin and eosin were used for counterstain.

© 2019. Song HD et al. Published online at http://www.aginganddisease.org/EN/10.14336/AD.2019.0810

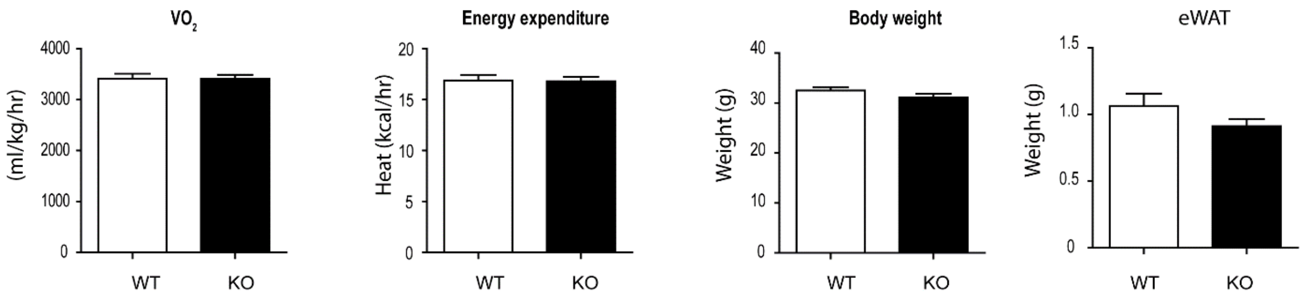

Supplementary Figure 15. Indirect calorimetry analysis, body weight and eWAT mass of 18-month-old BDNF^{Pdgfra} KO mice and WT control mice. There was no statistical difference between KO and WT mice. ($p = 0.968$ for VO_2; $p = 0.9008$ for Energy expenditure; $p = 0.1843$ for Body weight; $p = 0.1969$ for eWAT mass, $n = 6$).

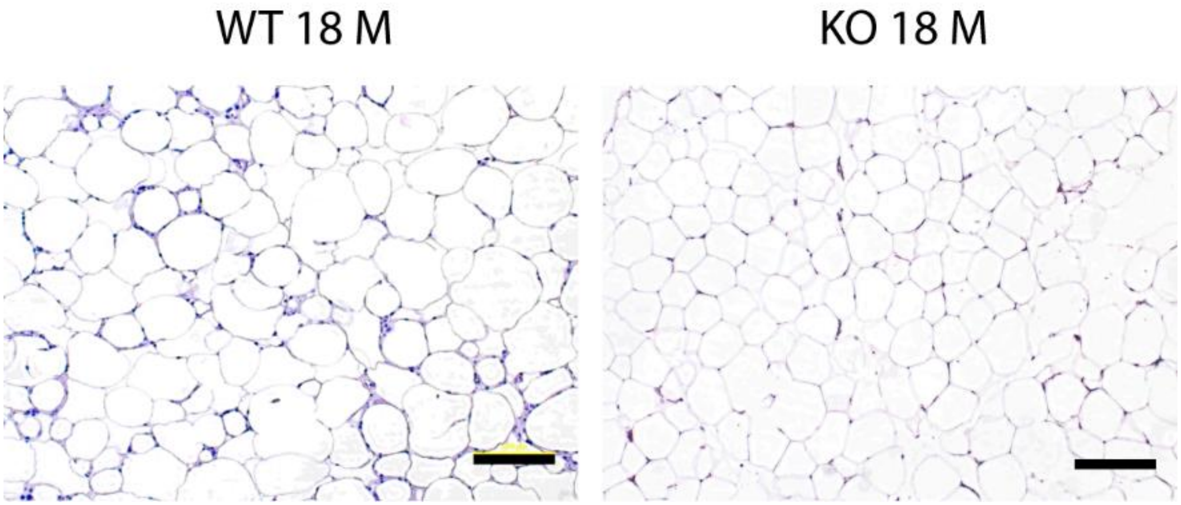

Supplementary Figure 16. Hematoxylin/eosin staining of paraffin sections of eWAT of 18-month-old BDNF^{Pdgfra} KO mice and WT control mice. Size bars = 100μm.

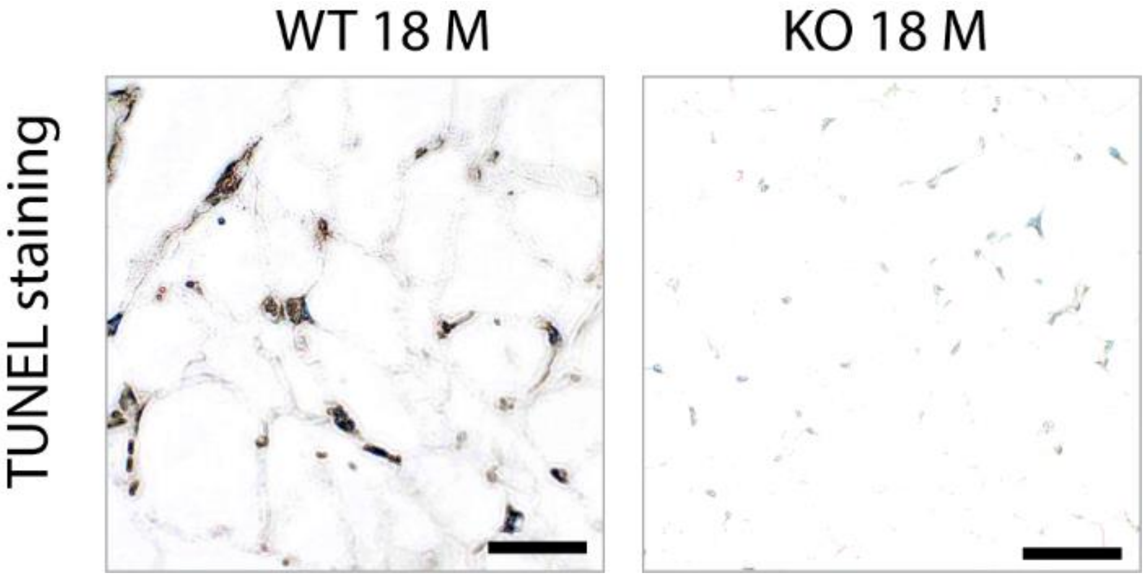

Supplementary Figure 17. TUNEL staining of paraffin sections of eWAT of 18-month-old BDNF^{PDGFRA}KO mice and control mice (Size bar = 50μm). Sections were counter-stained with Methyl Green.

© 2019. Song HD et al. Published online at http://www.aginganddisease.org/EN/10.14336/AD.2019.0810

SUPPLEMENTARY DATA

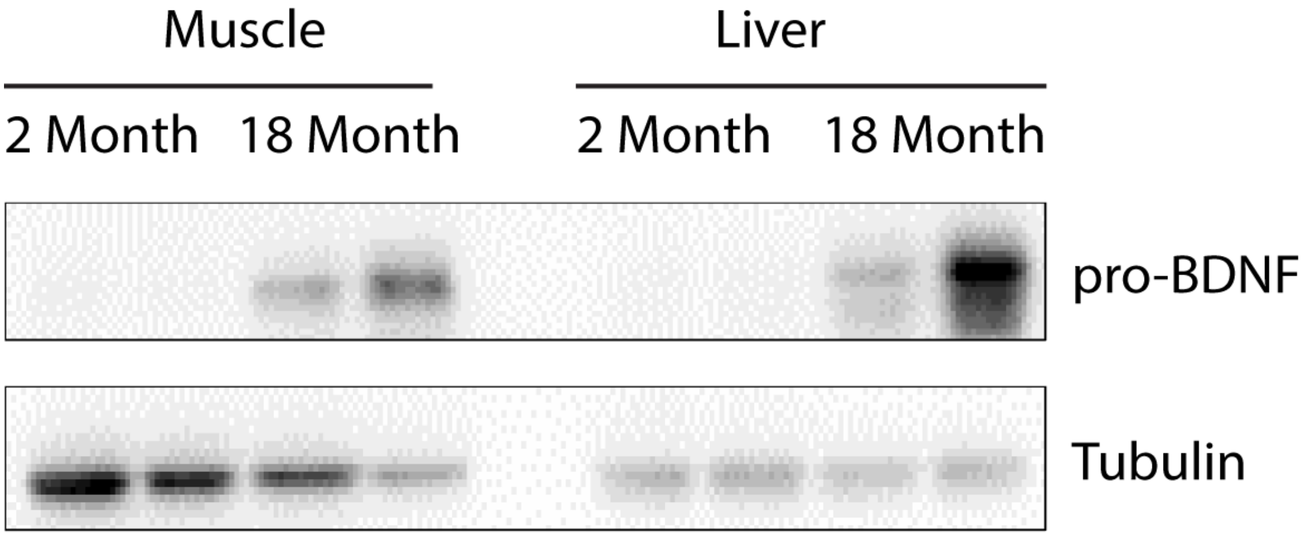

Supplementary Figure 18. Immunoblot analysis of pro-BDNF expression in liver and quadriceps muscle of mice.

© 2019. Song HD et al. Published online at http://www.aginganddisease.org/EN/10.14336/AD.2019.0810
